# A novel framework for intelligent surveillance system based on abnormal human activity detection in academic environments

**DOI:** 10.1007/s00521-016-2363-z

**Published:** 2016-06-03

**Authors:** Malek Al-Nawashi, Obaida M. Al-Hazaimeh, Mohamad Saraee

**Affiliations:** Al-Balqa’ Applied University, Irbid, Jordan

**Keywords:** Surveillance system, Abnormal activity detection, OMEGA equation, Support vector machines (SVM), MATLAB programming, Computer simulation

## Abstract

Abnormal activity detection plays a crucial role in surveillance applications, and a surveillance system that can perform robustly in an academic environment has become an urgent need. In this paper, we propose a novel framework for an automatic real-time video-based surveillance system which can simultaneously perform the tracking, semantic scene learning, and abnormality detection in an academic environment. To develop our system, we have divided the work into three phases: preprocessing phase, abnormal human activity detection phase, and content-based image retrieval phase. For motion object detection, we used the temporal-differencing algorithm and then located the motions region using the Gaussian function. Furthermore, the shape model based on OMEGA equation was used as a filter for the detected objects (i.e., human and non-human). For object activities analysis, we evaluated and analyzed the human activities of the detected objects. We classified the human activities into two groups: normal activities and abnormal activities based on the support vector machine. The machine then provides an automatic warning in case of abnormal human activities. It also embeds a method to retrieve the detected object from the database for object recognition and identification using content-based image retrieval. Finally, a software-based simulation using MATLAB was performed and the results of the conducted experiments showed an excellent surveillance system that can simultaneously perform the tracking, semantic scene learning, and abnormality detection in an academic environment with no human intervention.

## Introduction

**Fig. 1:**
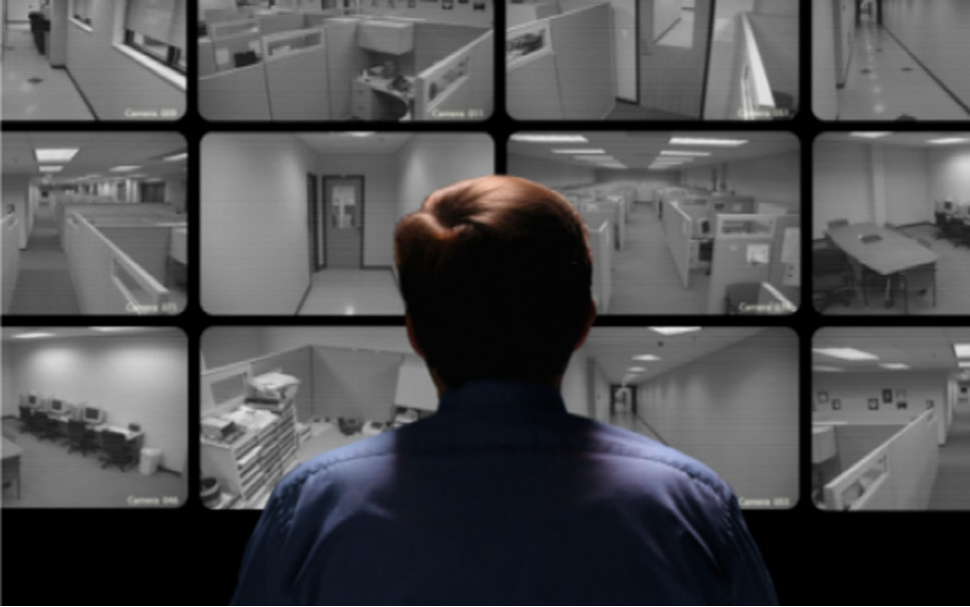
Traditional surveillance systems (control room)

Cameras attached to monitor screens are generally a traditional video surveillance system. A limited number of operators are responsible to constantly monitor a large area with the help of the cameras installed in various places as shown in Fig. 1 [1, 2]. When any unwanted incident happens, the operators warn the security or police. While some monitors show a video stream of a single camera, in other instances, a single monitor can show multiple streams simultaneously or sequentially [2].

But, in a few areas, the screens are not observed continually. The output of every camera is recorded by the video recorders. If there is an incident, the video footage can be utilized as proof. One weakness of this methodology is that operators are not ready to counteract the incidents or limit their harm because the recordings are only watched afterward. Another limitation is that it requires a lot of time to search for the right video pictures, particularly when the suspect is at the scene long before the incident takes place and when there are many cameras involved [3–5]. Because of these limitations, there is a need for a technique or method that can automatically detect and analyze human activities.

Over the last 10 years, there has been an increased attention to modern video surveillance in the wider community of computer vision. However, today, the visual surveillance community has a more focused attention to automated video surveillance system [6, 7], which is a network of video sensors that can observe human and non-human objects in a given environment. The system can analyze patterns of normal/abnormal activities, interesting events, and other designated activities or goals. However, due to varying weather conditions where the video surveillance systems have to operate and given that the systems have to work all the time, robust detection and tracking of objects in the systems become more important. In these situations, a minimal margin of error is expected of the systems [1, 8, 9]. This paper describes the development of an intelligent surveillance system for abnormal human activity in an academic environment. The proposed surveillance system incorporates a wide range of advanced surveillance techniques: real-time moving object detection, tracking from stationary camera platforms, recognition of generic object classes and specific human abnormal behavior, and triggering an alarm.

**Fig. 2:**
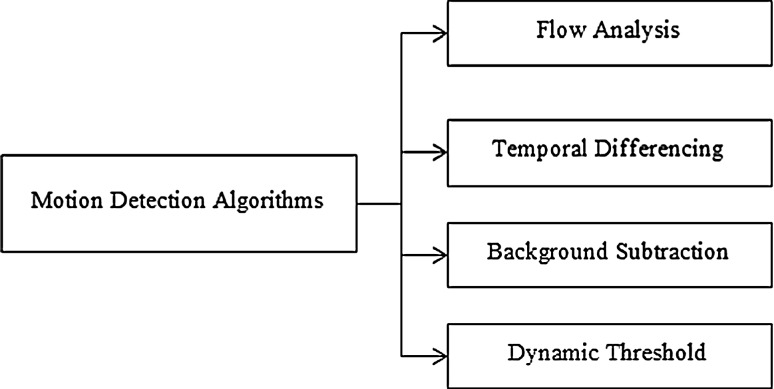
Motion detection algorithms

Detecting moving objects and compressing their image are low-level tasks that are commonly used in many applications of computer vision, such as surveillance, monitoring, robot technology, and object recognition, to name a few [10–12]. A number of methods have been suggested to detect moving objects and compress their images, especially in relation to human and visual surveillance. There are four major categories of algorithms for motion detection. They are background subtraction, temporal differencing, flow analysis, and dynamic threshold. These categories are shown in Fig. 2 [13, 14]. Our approach is based on temporal differencing and a flow analysis for abnormal human activity detection.

Some of the relevant works in the field of motion detection and image compression are mentioned in the following section.

## Related work

In this section, we report a survey on the techniques and methods relevant to motion detection, specifically, approaches to detecting a moving object. For accurate detection, the motion must be accurately detected using suitable methods. Many researchers have turned their attention to proposing new methods for motion detections, but the new methods have a number of practical problems, such as shadow and lighting change over time.

Ansari et al. [15] proposed a motion detection system that provides an efficient method for surveillance purposes and provides the user a facility to use an audio file as an alarm signal. Augustin et al. [16] focused on the tracking method to detect the moving object. The method is simple and direct in which the changing part in the video can be quickly detected. Hati et al. [17] proposed a new temporal-differencing method to detect a moving object. This method is fast and achieves better detection performance in terms of triggering an alarm on time with high accuracy and has a very low false alarm.

Motion detection and object tracking [18] is a popular technique which is robust against the complex, deformed, and changeable shape. This method is scale and rotation invariant, as well as faster in terms of processing time. Antonakaki et al. [19] proposed a new temporal-differencing approach which is robust in which statistical activity recognition is used for modeling activities.

Foresti et al. [9] used the theory of segmentation to propose a new method to detect with high accuracy the moving object inside the monitored scene. Elarbi-Boudihir and Al-Shalfan [12] described a new surveillance system that consumes low power because the motion detection approach reduces the unwanted recording of surveillance videos. Foresti et al. [20] used a background subtraction technique to detect the moving object and then remove the shadow in the subsequent phase.

To obtain the change region, Gupta and Sawarkar [21] employed a change detection method that has low computational load and system complexity, to analyze temporal information between successive frames. To detect objects more accurately from the input image, Dhar et al. [22] utilized a motion detection-based approach that has a manual threshold selection. A background subtraction method was proposed by Lee et al. [23] that can effectively extract motion objects. The method is less sensitive to illumination changes.

## Proposed approach

**Fig. 3:**
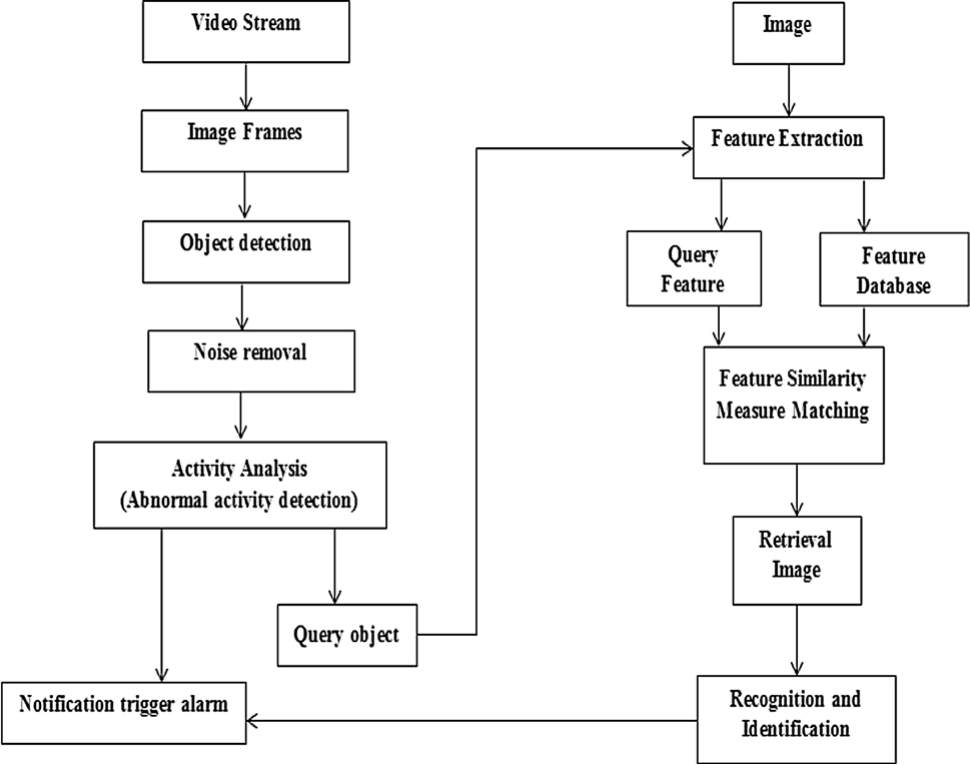
Flow diagram of our proposed system architecture

This paper proposes a new intelligent surveillance system for human monitoring and visual surveillance to reduce human efforts (i.e., in a control room). The proposed surveillance system was conducted in three phases: Preprocessing phase, abnormal activity detection phase, and content-based image retrieval phase. An overview of the flow diagram of our proposed intelligent surveillance system architecture is shown in Fig. 3. In this section, we discuss the detailed implementation phases of the proposed approach.

### Preprocessing phase

In this phase, all students must register before commencing a course of study at the university. Registration refers to a formal process whereby a student enrolls at the start of his/her period of study to become part of the student community. The registration consists of several stages. The two most important stages are used in our surveillance system. The first stage involves collecting personal details (i.e., the first name, the middle name, the family name, nationality, program name, birth date, identification card number.). The second stage is the photographing stage. At this stage, all students are required to submit a photo of them to generate a student card. The proposed system also requires students to submit their own photo in different situations (i.e., fear, anger, sadness, displeasure, and surprise) to get an accurate description in terms of content-based image retrieval.

In the proposed intelligent surveillance system, both of these records are stored in a database for content-based image retrieval (CBIR) in case the proposed system detects an abnormal student’s activities.

### Abnormal activity detection phase

**Fig. 4:**
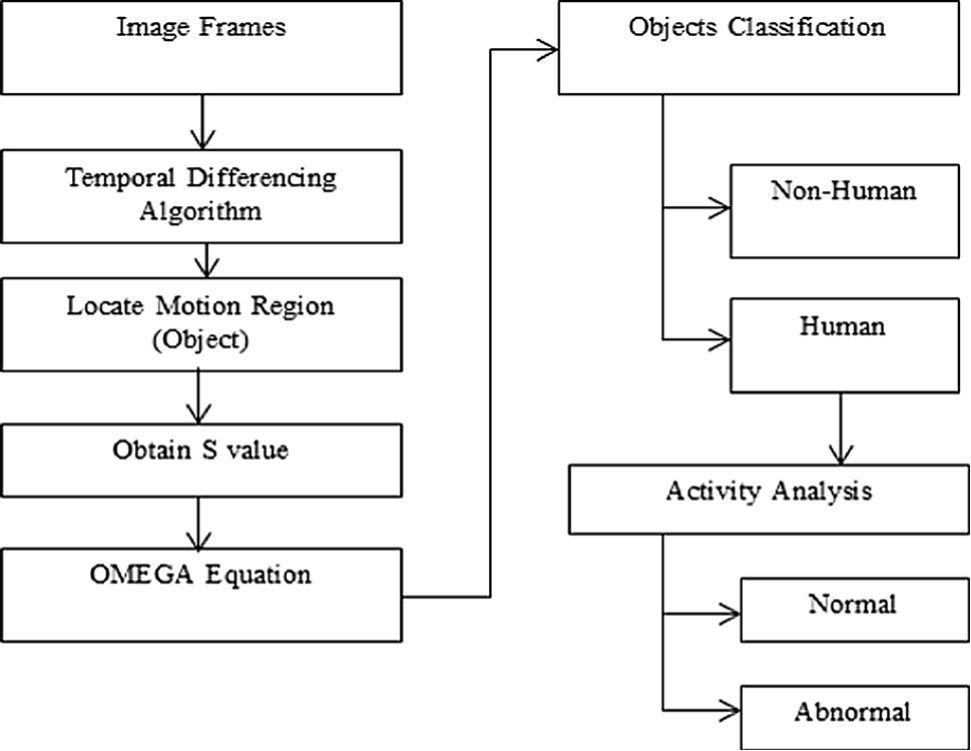
Flow diagram of object detection

In this phase, a set of devices are used to monitoring and capturing a video stream. For image framing, the video must be divided into a sequence of frames mostly into 25–30 frames [5], which are then sent to the next step (i.e., object detection) for further processing. The flow diagram for this phase is shown in Fig. 4.

**Fig. 5:**
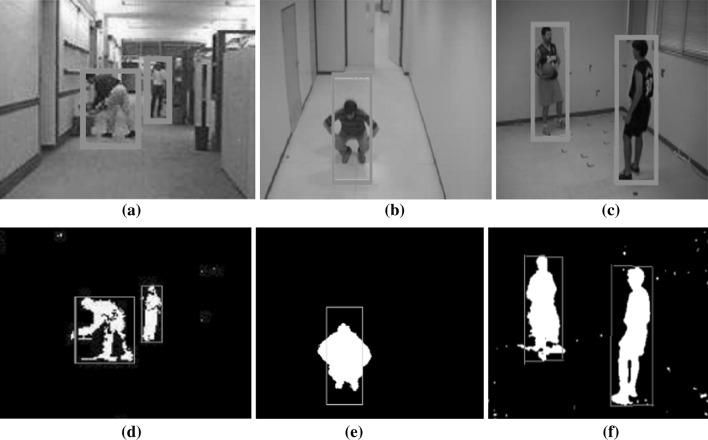
Moving object and detected motion regions

As mentioned earlier, there are three main conventional approaches to object detection: background subtraction, temporal difference, optical flow, and dynamic threshold as shown in Fig. 2. In our system, moving objects are detected in a video stream using the temporal-differencing algorithm. Then, the motion region is located by frame tracking as shown in Fig. 5. The video captures a module, which is delivered to a video stream acquired from the camera. Then, each frame of the stream is smoothed with the second derivative in time of the temporal Gaussian function based on absolute difference function $\Delta n$ as shown in Eq. 1. Then, a motion image *M*_*n*_ can be extracted using the threshold function as shown in Eqs. 2 and 3 [12].

1 $$\Delta n = abs\left( {f_{n} - f_{n - 1} } \right)$$

2 $$M_{{n \left( {u,v} \right) = }} f_{n} \left( {u,v} \right) ,\quad{\text{if}}\,\Delta n\left( {u,v} \right) \ge T$$

3 $$M_{{n \left( {u,v} \right) = }} 0 ,\quad {\text{if}}\,\Delta n\left( {u,v} \right) < T$$

where *T* is an appropriate threshold chosen after several tests performed on the scenes of the environment. To separate the regions of interest from the rest of the image, binary statistical erosion and dilatation are used as shown in Eqs. 4 and 5, respectively.

4 $$f_{e} \left( i \right) = \left\{ {\begin{array}{*{20}l} {1 , \quad M^{1} \left( i \right) \ge T} \hfill \\ {0 , \quad M^{1} \left( i \right) < T} \hfill \\ \end{array} } \right.$$

5 $$f_{d} \left( i \right) = \left\{ {\begin{array}{*{20}l} {1 ,\quad {\text{if}}\,M^{1} \left( i \right) \ge 1} \hfill \\ {0 ,\quad {\text{if}}\,M^{1} \left( i \right) < 1} \hfill \\ \end{array} } \right.$$

To remove noise, binary statistical erosion can eliminate the noisy isolated pixels. Then, binary statistical dilatation allows the interesting pixels eliminated by erosion to recover. After the motion region is determined, the moving objects are clustered into a motion region using a connected component criterion. The motion region (i.e., motion region box) that contains a person who has entered the field of view is located. The shape model can be used as a filter which ignores non-human objects based on the similarity to the shape model (i.e., unique pattern of *S*) as discussed in [24, 25]. In the proposed system, the shape model is used based on OMEGA equation to obtain the pattern of *S* as shown in Eq. 6:

6 $$Y = \sqrt {S^{2} - X^{2} + abs\left( X \right)} - \frac{abs\left( X \right)}{K} \cdot \sqrt {abs(S^{2} + X^{2} )}$$

To find the value of *S* for a given shape, let,

$$Q^{2} = S^{2} - X^{2}$$

Then, Eq. (6) can be written as,

$$Q = \frac{{\frac{ - 2\left| X \right| \cdot Y }{K} \pm \sqrt {\frac{{4X^{2 } Y^{2} }}{{K^{2} }} - \left( {\frac{{4X^{2} }}{{K^{2} }} - 4} \right)\left( {Y^{2} - abs\left( X \right)} \right)} }}{{\left( {\frac{{2X^{2} }}{{K^{2} }} - 1} \right)}}$$

Let,

$$m = X^{2} Y^{2} - \left( {X^{2} - Y^{2} } \right)\left( {Y^{2} - abs\left( X \right)} \right)$$

$$n = X^{2} - Y^{2}$$

Then, for the unique pattern of S, we get,

7 $$S = \sqrt {\frac{1}{{n^{2} }}\left[ {\left( {X^{2} Y^{2} + m^{2} \pm 2\left| X \right| \cdot Y \cdot m} \right) + X^{2} } \right]}$$

**Fig. 6:**
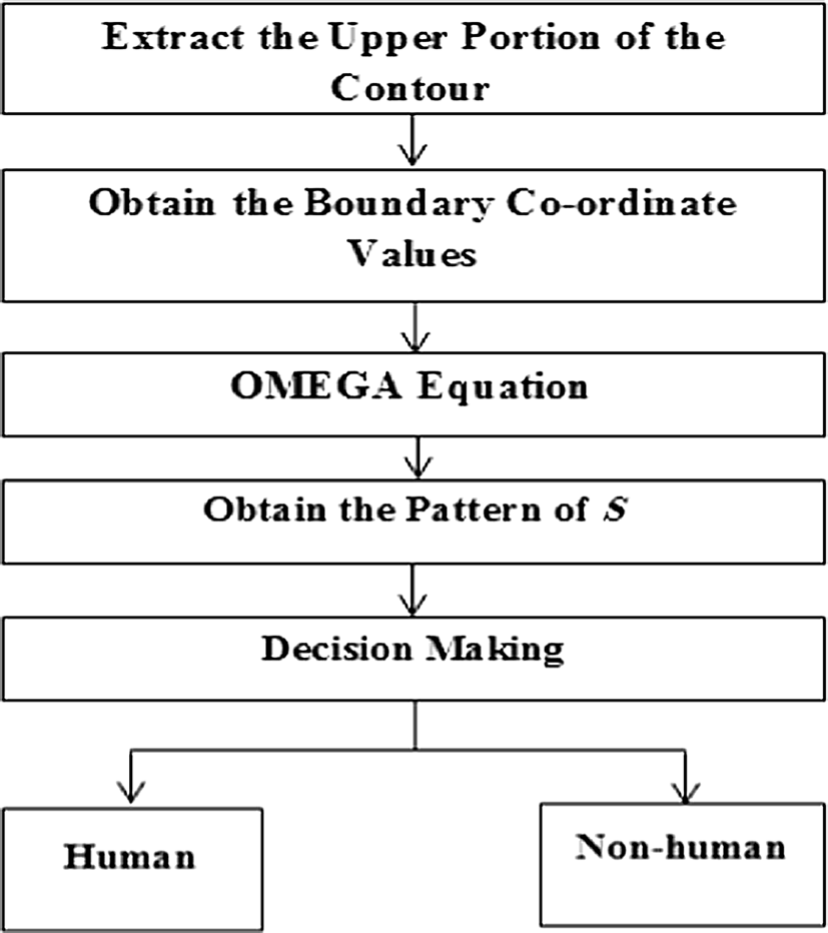
Flow diagram of shape model based on OMEGA equation

Figure 6 shows the flow diagram of the shape model which is implanted in the proposed surveillance system.

To make it clear, a shape model is based on a set of parallel and sequential steps, which are partially automated:

**Table:** 

Steps of the shape model based on OMEGA equation:	
Step I	The motion region box is designed to include the object of interest and whose axes are aligned with the image axes as shown in Fig. 5d–f.
Step II	Based on the set of boundary points obtained (i.e., Motion region box), coordinates (*C*_*x*_, *C*_*y*_) are calculated.
Step IV	Obtain the distance *d* = (*C*_*y*_ − *Y*_min_).
Step V	Obtain *H* = half of distance, where *H* is the window height for extracting the head and shoulder portion of the human object.
Step VI:	Extract the set of coordinates from the boundary of the upper-segmented contour.
Step VII	These values are then substituted in Eq. 7 to obtain the pattern for *S*.

**Fig. 7:**
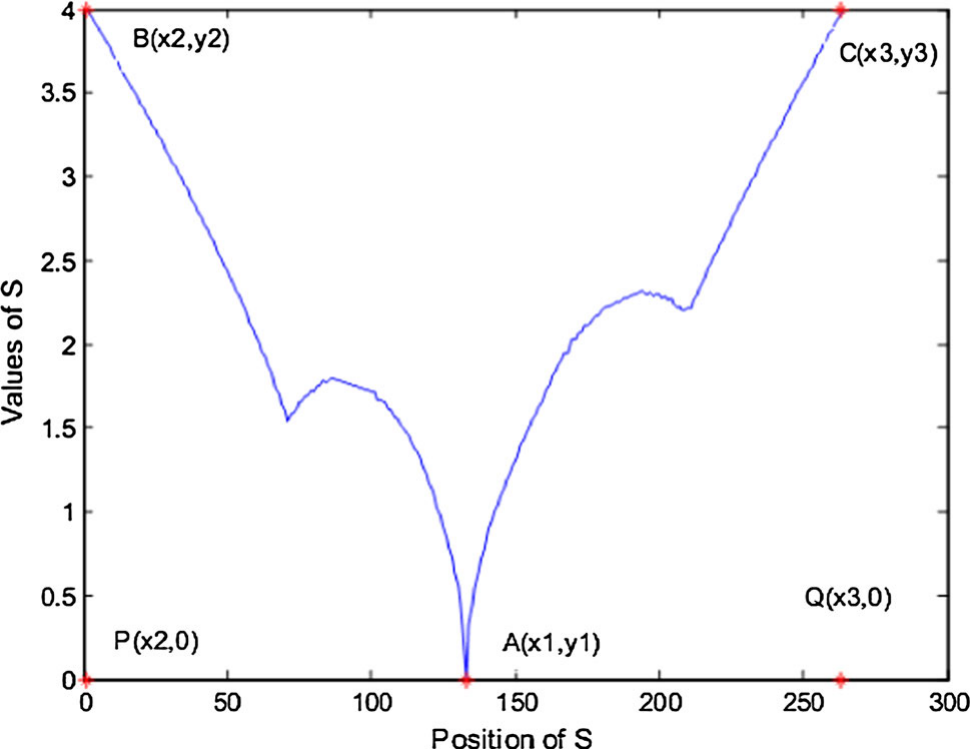
Typical pattern of *S* for human shape

Figure 7 shows a unique pattern of *S* for a human shape to classify the detected objects as human or non-human as discussed in [26].

**Fig. 8:**
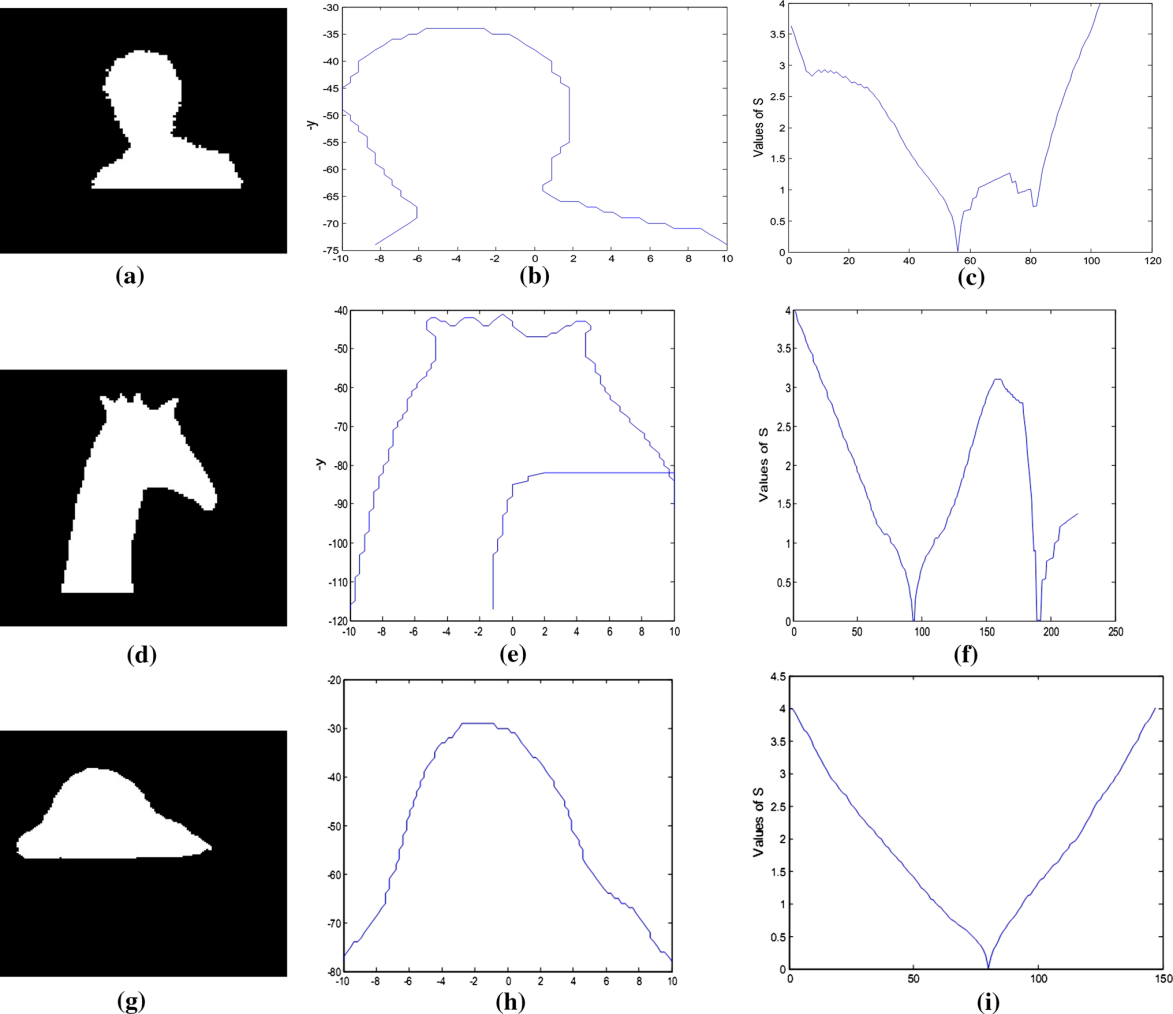
Experimental results using OMEGA equation. **a** Segmented contour, **b** coordinates, **c** pattern of S, **d** segmented contour, **e** coordinates, **f** pattern of S, **g** segmented contour, **h** coordinates, and **i** pattern of S

To make it clear, we have tested the typical pattern of *S* on a dataset containing some of human and non-human contours as shown in Fig. 8. The performed experiments were implemented through MATLAB application tool on a 1.6 GHz core i5 (IV), 8 GB memory, and 750 GB hard disk capacities, and the resolution of the camera is 320 × 240 QVGA. The success rate achieved is 97 %. Thus, it is very effective and robust in detecting human from images.

#### Human activity analysis

**Fig. 9:**
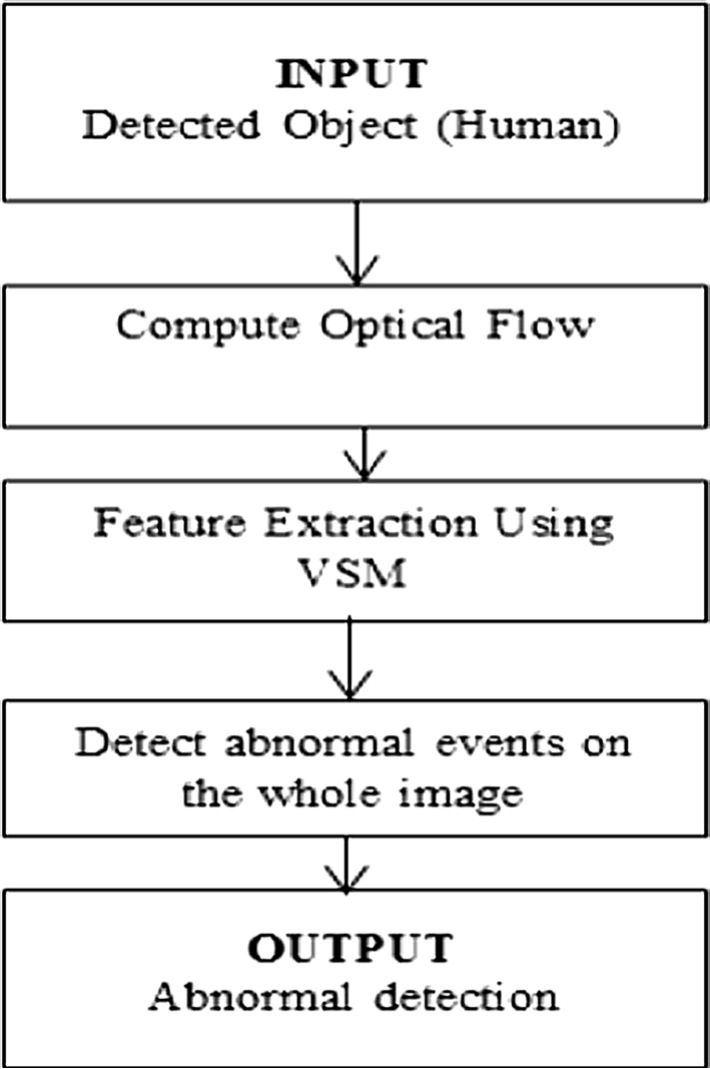
Flow diagram for human activity analysis

In this section, we proceed to evaluate and analyze the human activities as the detected objects and classify them into two groups: normal activities and abnormal activities, based on the support vector machine (SVM). The flow diagram for this step is shown in Fig. 9.

**Fig. 10:**
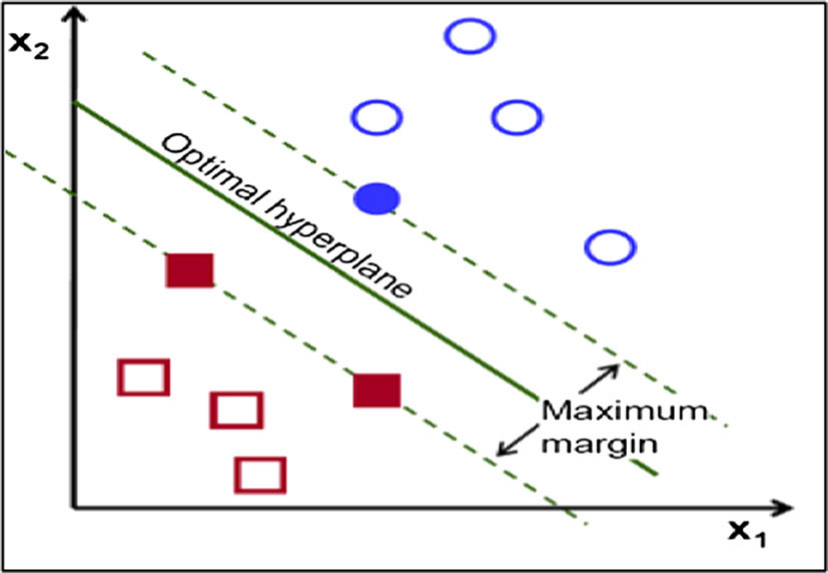
Maximum-margin HYPERPLANE for an SVM trained with samples from two classes

The basic idea of support vector machines (SVM) is to find the optimal HYPERPLANE that splits a dataset into different categories. Once the HYPERPLANE is chosen, the distance to the nearest data point of the classes is maximized [27]. Figure 10 gives an idea about a simple example with two classes in the plane.

Generally, support vector machine (SVM) is a discriminative classifier formally defined by a separating HYPERPLANE [28]. Equation 8 is used to define the HYPERPLANE:

8 $$f\left( x \right) = \beta_{0} + \beta_{x}^{T}$$

where *β* is known as the *weight vector* and *β*_0_ as the *bias*. The optimal HYPERPLANE can be represented in an infinite number of different ways by scaling of *β* and *β*_0_, the one of the possible ways to represent the optimal HYPERPLANE is shown in Eq. 9:

9 $$\left| {\beta_{0} + \beta_{x}^{T} } \right| = 1$$

where *X* symbolizes the training examples closest to the HYPERPLANE. Then, Eq. 10 gives the geometry distance between a point *X* and the optimal HYPERPLANE (*β*, *β*_0_):

10 $${\text{distance}} = \frac{{\left| {\beta_{0} + \beta_{x}^{T} } \right|}}{\beta }$$

**Fig. 11:**
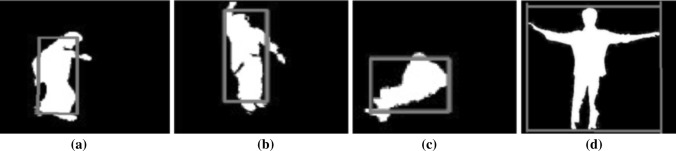
Detecting some abnormal activities. **a** Running: recognized when the speed of the frame from image to image goes beyond the threshold. **b** Jumping: recognized when the position of the frame from image to image abruptly goes up then down. **c** Falling: recognized when the size of the frame abruptly goes down relatively to the previous frame. **d** Flying: recognized when the size of the frame goes down, and up relatively to the previous frame

In the proposed system, using the geometry distance of the frame associated with the detected motion of the recognized object, we may categorize some basic activities like running, jumping, falling, and flying as shown in Fig. 11.

#### Alarm triggering

As mentioned in the previous sections, an abnormal activity can be any action such moving to any highly secure area, moving with speed more than a limit in a secure place, any typical pose that is not normal (i.e., falling and jumping), and many other actions which can trigger an alarm. Alarm triggering varies from customer to customer. It may include actually ringing any alarm, sending a notification to any department through e-mail or SMS, making an entry in the database, etc., to assist human operators to make the right decisions (i.e., warn the security or police).

### Content-based image retrieval phase

**Fig. 12:**
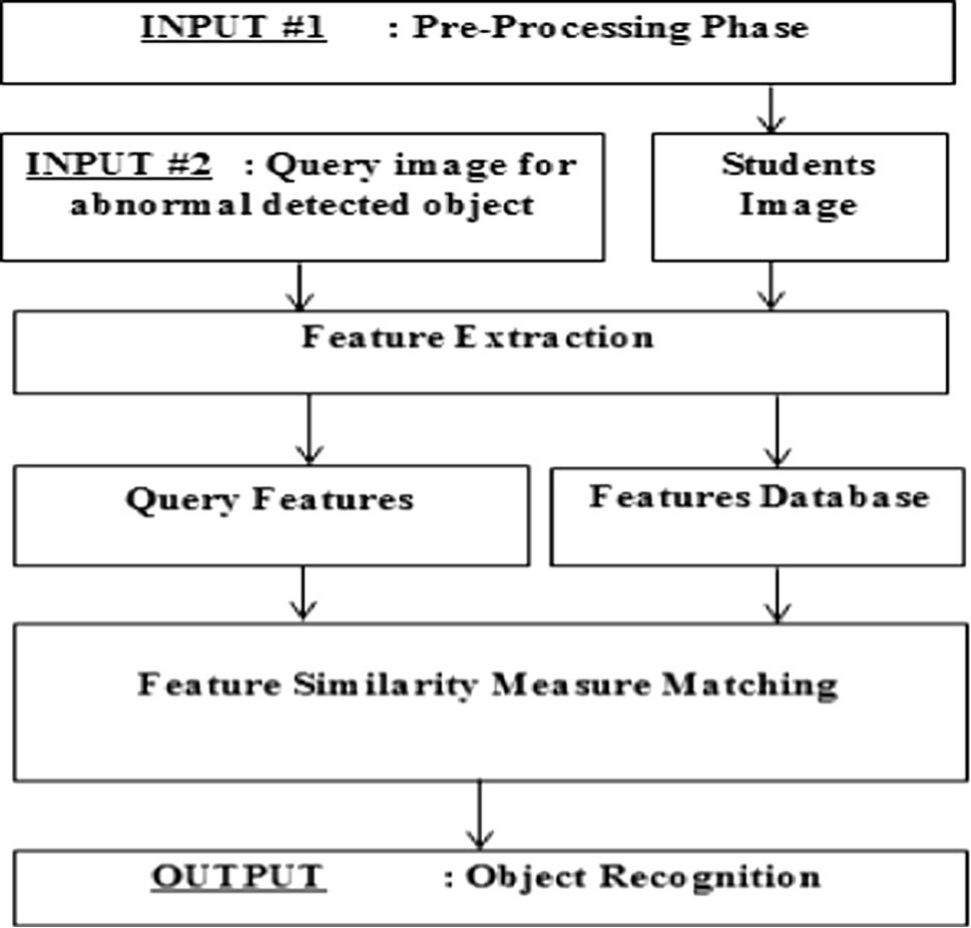
Flow diagram of content-based image retrieval

Content-based image retrieval (CBIR) in various computer vision applications is widely used to retrieve the desired images from a large collection on the basis of features that can be automatically extracted from the images themselves [29]. Figure 12 shows the flow diagram of a typical CBIR system which is implanted in the proposed surveillance system.

For each image in the image database (i.e., a student’s image), its features are extracted and the obtained feature space (or vector) is stored in the feature database. When a query image (i.e., abnormal detected object) comes in, its feature space will be compared with that in the feature database one by one and the similar images with the smallest feature distance will be retrieved (i.e., object recognition and identification) [30]. CBIR can be divided into the following stages: Preprocessing stage which involves filtering, normalization, segmentation, and object identification, and feature extraction stage such as shape, texture, and color as discussed and implemented in [31].

## Conclusion

In this paper, an automatic real-time video-based surveillance system in an academic environment for abnormal human behavior is proposed. We have divided the work into three phases: preprocessing phase, abnormal human activity detection phase, and content-based image retrieval phase. The proposed surveillance system is based on a flow analysis, temporal differencing, and threshold to detect abnormal human activities. For motion object detection, we used the temporal-differencing algorithm and then located the motions region using the Gaussian function. Furthermore, the shape model based on OMEGA equation is used as a filter for the detected objects, which ignores non-human objects based on their similarity to the shape model. For the object activities analysis, we evaluate and analyze the human activities for the detected objects and classify them into two groups: normal activities and abnormal activities, based on the support vector machine (SVM). The machine then provides an automatic warning in case of abnormal human activities. It is embedded with a method to retrieve the detected object from the database for object recognition using content-based image retrieval (CBIR). Finally, our propose system has been implemented through MATLAB application tools and the experiment results of the software simulation demonstrate the effectiveness of our proposed system, which can be considered a high-quality alternative to the other systems because of the high level of accuracy and performance and a very low false alarm.
